# Clustering of Poor Dietary Habits among Adolescents Aged 12 to 15 Years in 52 Low-Income and Middle-Income Countries

**DOI:** 10.3390/ijerph17186806

**Published:** 2020-09-18

**Authors:** Hui Fan, Xingyu Zhang

**Affiliations:** 1 Department of Preventive Medicine, North Sichuan Medical College, Nanchong 637000, China; 2 Applied Biostatistics Laboratory, University of Michigan School of Nursing, Ann Arbor, MI 48109, USA; zhangxyu@med.umich.edu

**Keywords:** poor dietary habits, adolescents, low- and middle-income countries

## Abstract

Very few studies have reported the co-occurrence of poor dietary habits. We thus aimed to estimate the co-occurrence of poor dietary habits in adolescents in low-income and middle-income countries (LMICs). Data were obtained from the Global School-Based Student Health Surveys (GSHS) from 2009 to 2017. The suboptimal dietary factors included fast food consumption, carbonated soft drink consumption, and low fruit and vegetable intake, which were assessed with a questionnaire survey. We calculated the corresponding country-specific prevalence with the number of suboptimal dietary factors. We also calculated pooled estimates across countries using a meta-analysis with random-effects. Our study included 145,021 adolescents between 12 and 15 years of age from 52 LMICs. The prevalence of fast food consumption, carbonated soft drink consumption, and low fruit and vegetable intake ranged from 20.9% in Pakistan to 80.0% in Thailand, from 22.4% in Kiribati to 79.3% in Suriname, and from 45.9% in Vanuatu to 90.7% in Nepal, respectively. The prevalence of exposure to two or three suboptimal dietary factors varied greatly across countries, ranging from 31.8% in Pakistan to 53.8% in Nepal and from 8.6% in Vietnam to 36.4% in Suriname, respectively. The pooled prevalence of exposure to two or three suboptimal dietary factors was 41.8% and 20.0%, respectively. Our findings indicate that poor dietary habits are frequent and tend to co-occur in adolescents in LMICs. Country-specific policies and programs are needed to address these conditions.

## 1. Introduction

Poor dietary habits continue to be a public health crisis because they play an important role in the onset of obesity, cardiovascular disease, diabetes, cancer, and mental disorders [1,2,3,4,5,6]. They also account for one of every five deaths globally [7]. It is well established that dietary behaviors in early life track into adulthood [8], so the establishment of healthy dietary behaviors early in life is crucial in the prevention of chronic non-communicable diseases and their association with premature death [9].

Fast food consumption, carbonated soft drink consumption, and low fruit and vegetable intake in children and adolescents have received considerable attention. Global surveys have shown that these three suboptimal dietary factors are frequent in adolescents [10,11], but adolescents in high-income countries have experienced improvements in their diet quality in recent years [12,13]. In contrast, due to the high cost of a healthy diet, adolescents in low-income and middle-income countries (LMICs) may tend to reduce their intake of healthy foods and become reliant on convenience food [12]. Studies have indicated that fast food consumption, carbonated soft drink consumption, and low fruit and vegetable intake in children and adolescents tend to co-occur [14,15]. However, the current co-occurrence of these three poor dietary habits remains unclear. We therefore sought to assess this issue in adolescents in LMICs using the Global School-Based Student Health Surveys (GSHS).

## 2. Materials and Methods

### 2.1. Study Population

The GSHS is a multi-country, cross-sectional, school-based survey used to assess behavioral risk factors in adolescents [5,6,10,11,16]. It was initiated by the World Health Organization (WHO) and the U.S. Centers for Disease Control and Prevention. The design and methods of the GSHS have been described in detail [5,6,10,11,16]. In brief, in each participating country, a two-stage cluster design was used to obtain a nationally representative sample, and a self-administered questionnaire survey was conducted to collect data on health behavior. Ethical approval was obtained from the Ministry of Education or Health and an institutional review board or ethics committee in each country. The participants and their parents provided verbal or written consent. Our study used data from the GSHS 2009–2017, which were publicly available at www.who.int/chp/gshs and www.cdc.gov/gshs, and exempt under the ethical board review of the corresponding author’s institution.

LMICs were based on the World Bank classification at the time of the survey for the respective countries. Public GSHS datasets were available from 75 LMICs. We excluded 23 country-specific datasets due to a lack of information regarding fast food consumption, carbonated soft drink consumption, or fruit or vegetable intake. We selected only the most recent survey if several GSHS waves were performed in a country. We also included only 12–15-year-old adolescents because data for this exact age range were available. Our study included 150,838 adolescents between 12 and 15 years of age. We then excluded 5817 adolescents with missing data for sex, food security, fast food consumption, carbonated soft drink consumption, fruit or vegetable intake. As a result, the final analysis sample consisted of 145,021 adolescents between 12 and 15 years of age from 52 LMICs (Figure S1).

### 2.2. Definitions

Sex, age, food security, fast food consumption, carbonated soft drink consumption, and low fruit and vegetable intake were assessed with a self-administered questionnaire. Adolescents who had eaten food from a fast food restaurant (country-specific examples, such as McDonalds or Burger King in Argentina) at least 1 day during the past 7 days were considered to be consumers of fast food [5]. Adolescents who had consumed carbonated soft drinks at least once per day for the past 30 days were considered to be consumers of carbonated soft drinks [10]. Low vegetable and fruit intake was defined as consumption of fewer than five servings of fruit and vegetables per day over the past 30 days [6,16]. The number of individual suboptimal dietary factors (fast food consumption, carbonated soft drink consumption, and low fruit and vegetable intake) were calculated.

### 2.3. Statistical Analysis

To account for the multistage sampling design, we used sampling weights to estimate the corresponding proportion and 95% confidence intervals (CIs) for each suboptimal dietary factor in each country. In addition, to evaluate the co-occurrence of suboptimal dietary factors in each country, we calculated the corresponding proportion and 95% CI with the number of suboptimal dietary factors (range, 0 to 3). We performed a meta-analysis with a random-effects model to compute the overall estimates. We also used the I^2^ statistic to evaluate heterogeneity among the country-specific estimates. We defined a high degree of heterogeneity with an I^2^ value of at least 75%. We performed subgroup analyses stratified by survey year (2009–2013 vs. 2014–2017), WHO region, and food security (yes vs. no). SAS version 9.4 (SAS Institute, Cary, NC, USA) and STATA version 11.0 (Stata Corporation, College Station, TX, USA) were used to conduct the statistical analyses.

## 3. Results

Our study included 145,021 adolescents between 12 and 15 years of age from 52 LMICs. Table 1 summarizes the characteristics of the participants across countries. The 52 LMICs were classified into five WHO regions (Americas, 14; Western Pacific, 13; Africa, 9; Eastern Mediterranean, 9; and Southeast Asia, 7). The survey year ranged from 2009 to 2017. The overall response rate was 96.1%, ranging from 87.2% in Liberia in 2017 to 99.1% in Peru in 2010. The sample sizes varied from 472 in Liberia in 2017 to 20,596 in Argentina in 2012.

Table 2 presents the prevalence of fast food consumption, carbonated soft drink consumption, and low fruit and vegetable intake. These data varied greatly across countries, ranging from 20.9% in Pakistan to 80.0% in Thailand, from 22.4% in Kiribati to 79.3% in Suriname, and from 45.9% in Vanuatu to 90.7% in Nepal, respectively. The pooled prevalence of fast food consumption, carbonated soft drink consumption, and low fruit and vegetable intake were 52.7%, 50.3%, and 74.4%, respectively.

Table 3 presents the co-occurrence of unhealthy eating habits across countries. The prevalence of exposure to two or three suboptimal dietary factors varied substantially across countries, ranging from 31.8% in Pakistan to 53.8% in Nepal and from 8.6% in Vietnam to 36.4% in Suriname, respectively. The pooled prevalence of exposure to two or three suboptimal dietary factors were 41.8% and 20.0%, respectively. The incidence of exposure to two suboptimal dietary factors exceeded 41.8% in 31 (59.6%) of the 52 participating countries. The incidence of exposure to all three suboptimal dietary factors exceeded 20.0% in 27 (51.9%) of the 52 participating countries. Table S1 further presents the co-occurrence of unhealthy eating habits stratified by survey year (2009–2013 vs. 2014–2017), WHO region (Americas, Western Pacific, Africa, Eastern Mediterranean, and Southeast Asia), and food security (yes vs. no). The relative high incidence of exposure to two or three suboptimal dietary factors can be observed by subgroup analyses. We also noted substantial heterogeneity in the prevalence estimates of the suboptimal dietary factors (Table 3 and Table S1; all I^2^ > 75%).

## 4. Discussion

Our findings indicate that suboptimal dietary factors, especially low fruit and vegetable intake, are frequent across LMICs and that suboptimal dietary factors tend to co-occur in adolescents despite the wide variations in their prevalence across countries.

Population-based studies have confirmed the adverse health consequences of fast food consumption, carbonated soft drink consumption, and low fruit and vegetable intake [1,2,3,4,5,6]. Several hypotheses have been proposed to explain these associations. First, fast food consumption leads to increased intake of energy, fat, saturated fat, and sodium [14]. Second, carbonated soft drink consumption leads to excessive sugar intake [17]. Third, low fruit and vegetable intake result in an inadequate intake of vitamins, minerals, dietary fiber, plant sterols, and flavonoids [18]. The fast food and soft drink industries target children and adolescents as potential and main consumers [19]. As a result, a prominent epidemic of suboptimal dietary factors has been observed in adolescents [10,11].

In LMICs, a transition from traditional diets to a Western diet has been noted [20]. The GSHS and other surveys have reported a high prevalence of fast food consumption, carbonated soft drink consumption, and low fruit and vegetable intake in adolescents in most LMICs [10,11,21,22,23,24]. We used updated GSHS data and presented similar results. Observation studies have also indicated the co-occurrence of suboptimal dietary factors in children and adolescents, but such co-occurrence in adolescents in LMICs remained unclear [14,15]. Given the high cost of a healthy diet and the consequent reliance on fast food in some LMICs [12], it is necessary to clarify the aforementioned issue. Our study demonstrates that the pooled prevalence of exposure to at least two suboptimal dietary factors was 61.8% in adolescents in LMICs. Our subgroup analyses stratified by survey year, WHO region, and food security did not show significant differences. Our study also showed that the co-occurrence of suboptimal dietary factors in adolescents is prominent in most LMICs.

Our results have two key implications for the establishment of policies and programs to address suboptimal dietary factors. First, a comprehensive policy and program that includes high taxes and advertising ban for unhealthy fast food and carbonated soft drinks, a sufficient supply of inexpensive fruits and vegetables, health education, a key parental role, and behavioral interventions are needed given the co-occurrence of suboptimal dietary factors [25,26,27,28,29]. Second, the wide variations in the co-occurrence of suboptimal dietary factors and the substantial heterogeneity in the prevalence estimates across countries indicate that country-specific policies and programs for LMICs should be developed. The effective policies and programs are based on the social norms, cultural influences, and the context of poor dietary habits [5,10,16]. In addition, although the co-occurrence of suboptimal dietary factors is a universal phenomenon, we also note that a few adolescents (pooled estimate: 4.6%) in LMICs follow the principles of healthy eating. The reasons for developing healthy eating habits are needed to clarify how to help countries establish effective policies and programs.

The strengths of our study include its nationally representative sample and comparable country-specific results due to the standard procedures used for data collection. However, our study has several limitations. First, our study includes no countries from Europe. Second, some countries may not have a sufficient sample size to estimate the co-occurrence of suboptimal dietary factors. Third, the study population was restricted to adolescents who attend school. Fourth, the suboptimal dietary factors are self-reported, which may have introduced bias. Fifth, the GSHS data for 52 LMICs were collected over a long time period (2009–2017). Direct comparisons between countries may have introduced bias due to the difference in the survey year. However, subgroup analyses stratified by survey year were performed between a narrow time interval (2009–2013 or 2014–2017), and we also observed the high incidence of exposure to two or three suboptimal dietary factors (Table S1). Of note, surveys for several countries were conducted before 2013. High-quality surveillance data about behavioral risk factors from GSHS were important in several LMICs where relevant data were scarce [16]. Consequently, we kept the country-specific data from the GSHS which were conducted before 2013. Given the transition from traditional diets to a Western diet in LMICs, the updated data for these countries were needed to assess trends over time and establish country-specific policies and programs. Sixth, the GSHS did not involve the clear definition of fast food. The relevant result should be interpreted with caution. Finally, we observed substantial heterogeneity in the co-occurrence of suboptimal dietary factors. Future studies should seek to explore the reasons for this heterogeneity.

## 5. Conclusions

In summary, our results suggest that poor dietary habits are frequent and tend to co-occur in adolescents in LMICs. Country-specific policies and programs are needed to address these conditions given the wide variations in their prevalence across countries.

## Figures and Tables

**Table 1:** Characteristics of all participants aged 12–15 years across countries.

Country	Survey Year	Region	Response Rate, %	N	Mean Age, Years	Boys, %
Afghanistan	2014	Eastern Mediterranean	88.3	1319	14.1	53.6
Algeria	2011	Africa	97.8	3408	13.6	45.8
Argentina	2012	America	95.7	20,596	13.9	47.6
Bangladesh	2014	South-East Asia	94.3	2597	14.0	63.8
Belize	2011	America	97.4	1559	13.6	48.0
Benin	2016	Africa	97.6	700	14.2	65.8
Bolivia	2012	America	95.6	2682	14.0	50.1
Cambodia	2013	Western Pacific	98.7	1789	14.1	48.5
Costa Rica	2009	America	98.1	2223	14.0	49.8
Dominica	2009	America	97.1	1272	13.6	49.9
Dominican Republic	2016	America	94.7	903	14.3	48.2
Egypt	2011	Eastern Mediterranean	94.4	2232	13.5	48.7
El Salvador	2013	America	96.6	1560	14.0	50.6
Eswatini	2013	Africa	97.0	1278	14.1	39.2
Fiji	2016	Western Pacific	92.2	1417	14.4	48.9
Ghana	2012	Africa	96.9	1298	13.9	48.8
Guatemala	2015	America	94.6	3417	13.9	50.4
Guyana	2010	America	95.8	1891	14.1	48.4
Honduras	2012	America	95.9	1425	13.6	46.2
Indonesia	2015	South-East Asia	97.5	8589	13.5	49.0
Iraq	2012	Eastern Mediterranean	96.2	1475	13.9	54.7
Jamaica	2017	America	95.7	1015	14.2	47.4
Kiribati	2011	Western Pacific	97.2	1302	14.0	45.3
Lao People’s Democratic Republic	2015	Western Pacific	98.6	1621	14.5	47.8
Lebanon	2017	Eastern Mediterranean	97.2	3254	13.6	47.1
Liberia	2017	Africa	87.2	472	14.0	50.8
Malaysia	2012	Western Pacific	99.0	16,106	14.0	49.5
Maldives	2014	South-East Asia	94.1	1676	14.4	48.8
Mauritania	2010	Africa	93.2	1197	14.2	53.4
Mongolia	2013	Western Pacific	97.1	3599	13.7	49.4
Morocco	2016	Eastern Mediterranean	92.4	3674	13.6	50.8
Mozambique	2015	Africa	88.8	593	14.1	51.1
Namibia	2013	Africa	94.9	1837	14.1	42.4
Nepal	2015	South-East Asia	95.3	4400	13.7	47.5
Pakistan	2009	Eastern Mediterranean	97.2	4860	14.1	60.7
Paraguay	2017	America	95.4	1882	13.9	47.4
Peru	2010	America	99.1	2338	14.1	49.8
Philippines	2015	Western Pacific	98.8	6088	13.9	48.1
Samoa	2011	Western Pacific	90.0	1991	14.0	47.1
Solomon Islands	2011	Western Pacific	91.7	848	14.1	51.9
Sri Lanka	2016	South-East Asia	97.3	2194	13.9	49.1
Sudan	2012	Eastern Mediterranean	93.6	1312	14.2	51.9
Suriname	2016	America	97.5	1416	13.8	46.0
Syrian Arab Republic	2010	Eastern Mediterranean	97.1	2843	13.6	50.7
Thailand	2015	South-East Asia	95.7	3956	13.7	49.3
Timor-Leste	2015	South-East Asia	90.1	1470	14.1	46.0
Tonga	2017	Western Pacific	97.0	2004	13.6	51.2
Tuvalu	2013	Western Pacific	93.5	635	13.3	48.2
Tanzania	2014	Africa	95.7	2502	13.6	47.0
Vanuatu	2016	Western Pacific	95.0	1224	14.1	47.3
Vietnam	2013	Western Pacific	97.4	1697	14.5	46.8
Yemen	2014	Eastern Mediterranean	89.2	1385	13.8	56.5

N = Sample size with complete data.

**Table 2:** Prevalence of fast food consumption, carbonated soft drink consumption, and low fruit and vegetable intake.

Country	Carbonated Soft Drink Consumption, % (95% CI)	Fast Food Consumption, % (95% CI)	Low Fruit and Vegetable Intake, % (95% CI)
Afghanistan	40.7 (36.0, 45.4)	63.4 (57.1, 69.6)	85.2 (80.6, 89.9)
Algeria	77.6 (75.2, 80.1)	51.7 (48.3, 55.0)	65.5 (62.6, 68.5)
Argentina	65.7 (63.5, 68.0)	31.3 (29.2, 33.3)	82.5 (81.0, 83.9)
Bangladesh	47.8 (43.8, 51.9)	53.5 (48.6, 58.3)	83.6 (79.7, 87.5)
Belize	63.8 (60.4, 67.1)	65.7 (60.8, 70.6)	70.5 (67.8, 73.3)
Benin	43.3 (37.3, 49.3)	46.8 (40.3, 53.2)	69.0 (63.5, 74.5)
Bolivia	63.3 (60.8, 65.7)	57.0 (54.5, 59.6)	68.4 (65.7, 71.1)
Cambodia	45.6 (40.6, 50.6)	25.4 (22.3, 28.6)	89.9 (88.3, 91.4)
Costa Rica	52.7 (49.0, 56.4)	54.4 (48.8, 60.1)	80.7 (78.0, 83.4)
Dominica	56.1 (52.7, 59.5)	46.4 (43.0, 49.8)	73.5 (70.1, 76.8)
Dominican Republic	74.9 (73.3, 76.6)	46.0 (40.9, 51.2)	81.1 (77.0, 85.2)
Egypt	55.2 (48.8, 61.6)	49.0 (42.5, 55.5)	75.2 (69.6, 80.8)
El Salvador	66.2 (62.4, 70.1)	57.0 (53.4, 60.5)	79.1 (76.5, 81.8)
Eswatini	45.7 (42.3, 49.1)	41.1 (37.0, 45.2)	79.6 (75.8, 83.4)
Fiji	63.3 (59.1, 67.5)	63.8 (58.4, 69.2)	62.7 (57.1, 68.4)
Ghana	53.8 (46.6, 61.0)	69.0 (60.8, 77.3)	65.1 (60.8, 69.3)
Guatemala	61.2 (55.6, 66.7)	57.2 (49.1, 65.4)	70.8 (68.7, 72.9)
Guyana	71.0 (66.5, 75.4)	55.5 (52.6, 58.5)	68.4 (63.6, 73.2)
Honduras	73.7 (70.2, 77.2)	47.5 (43.8, 51.2)	73.5 (69.7, 77.4)
Indonesia	28.9 (26.5, 31.4)	54.7 (52.1, 57.2)	75.3 (73.3, 77.3)
Iraq	53.9 (50.6, 57.2)	55.5 (50.8, 60.1)	73.4 (69.6, 77.1)
Jamaica	70.2 (65.3, 75.1)	58.6 (55.9, 61.3)	81.3 (77.1, 85.6)
Kiribati	22.4 (19.5, 25.4)	43.7 (39.4, 48.0)	85.4 (83.4, 87.4)
Lao People’s Democratic Republic	58.0 (50.2, 65.8)	44.5 (38.9, 50.1)	81.9 (79.3, 84.6)
Lebanon	48.8 (45.2, 52.4)	77.0 (75.0, 79.0)	75.6 (73.1, 78.2)
Liberia	47.3 (40.8, 53.8)	41.5 (34.7, 48.4)	71.3 (66.8, 75.8)
Malaysia	31.1 (29.4, 32.8)	48.3 (46.6, 49.9)	69.9 (68.6, 71.2)
Maldives	32.5 (29.4, 35.6)	34.6 (30.5, 38.6)	90.2 (88.5, 91.8)
Mauritania	52.2 (48.5, 55.8)	62.7 (56.7, 68.7)	71.4 (66.9, 75.8)
Mongolia	33.0 (30.4, 35.6)	55.2 (49.7, 60.7)	78.5 (76.8, 80.1)
Morocco	33.4 (31.4, 35.4)	61.8 (57.9, 65.7)	64.1 (60.3, 67.9)
Mozambique	57.7 (45.8, 69.7)	64.5 (53.5, 75.5)	75.4 (70.2, 80.6)
Namibia	51.5 (47.1, 55.9)	53.8 (47.9, 59.6)	71.4 (66.9, 75.9)
Nepal	33.4 (28.8, 38.0)	75.2 (71.2, 79.2)	90.7 (88.3, 93.1)
Pakistan	36.5 (29.7, 43.3)	20.9 (17.5, 24.3)	90.0 (87.9, 92.1)
Paraguay	61.3 (57.5, 65.0)	54.5 (50.3, 58.6)	73.1 (71.1, 75.2)
Peru	53.2 (49.5, 57.0)	50.1 (46.0, 54.1)	90.2 (87.4, 92.9)
Philippines	37.7 (34.7, 40.7)	51.9 (46.0, 57.8)	74.5 (72.4, 76.6)
Samoa	53.5 (50.3, 56.6)	78.8 (75.3, 82.3)	52.1 (48.2, 56.0)
Solomon Islands	43.8 (39.7, 47.9)	64.6 (54.3, 75.0)	55.2 (50.4, 60.0)
Sri Lanka	26.7 (23.0, 30.5)	42.7 (36.9, 48.5)	75.8 (72.7, 79.0)
Sudan	38.7 (34.2, 43.2)	41.2 (35.1, 47.3)	77.1 (73.7, 80.5)
Suriname	79.3 (76.5, 82.1)	63.9 (60.0, 67.8)	71.0 (68.2, 73.7)
Syrian Arab Republic	30.9 (27.7, 34.2)	42.7 (37.3, 48.1)	84.5 (82.7, 86.4)
Thailand	57.0 (51.5, 62.6)	80.0 (78.0, 81.9)	70.0 (66.7, 73.2)
Timor-Leste	44.2 (39.8, 48.7)	66.6 (62.6, 70.6)	83.5 (79.6, 87.4)
Tonga	60.7 (58.2, 63.1)	69.7 (67.5, 71.9)	54.8 (52.4, 57.2)
Tuvalu	54.0 (50.0, 57.9)	44.4 (40.6, 48.3)	64.9 (61.1, 68.5)
Tanzania	47.5 (42.5, 52.4)	35.3 (30.2, 40.5)	65.0 (60.3, 69.6)
Vanuatu	41.2 (37.3, 45.1)	57.3 (52.4, 62.1)	45.9 (41.8, 49.9)
Vietnam	34.9 (28.5, 41.3)	29.7 (25.8, 33.6)	77.1 (73.9, 80.2)
Yemen	37.0 (29.2, 44.9)	34.2 (25.3, 43.1)	79.1 (74.1, 84.2)
Pooled estimate	50.3 (45.6, 55.0)	52.7 (48.4, 57.0)	74.4 (71.8, 77.0)
I^2^ (%)	99.1	99.1	98.1

CI, confidence interval.

**Table 3:** Clustering of unhealthy eating habits across countries.

Country	Number of Unhealthy Eating Habits, % (95% CI)			
	0	1	2	3
Afghanistan	2.2 (0.7, 3.7)	29.2 (25.1, 33.3)	45.8 (41.1, 50.4)	22.9 (19.4, 26.3)
Algeria	2.7 (1.8, 3.5)	25.0 (23.0, 26.9)	47.2 (45.1, 49.2)	25.2 (22.4, 27.9)
Argentina	3.5 (2.9, 4.0)	31.8 (30.2, 33.3)	46.6 (45.5, 47.7)	18.2 (16.6, 19.7)
Bangladesh	1.4 (0.4, 2.5)	34.4 (29.8, 39.0)	41.9 (38.1, 45.6)	22.3 (19.0, 25.5)
Belize	4.0 (3.0, 5.0)	22.0 (18.5, 25.5)	44.0 (40.2, 47.8)	30.0 (26.8, 33.2)
Benin	5.2 (3.0, 7.4)	43.5 (38.9, 48.1)	38.3 (33.9, 42.6)	13.0 (10.5, 15.5)
Bolivia	5.2 (4.3, 6.1)	26.8 (25.4, 28.2)	42.2 (40.6, 43.7)	25.9 (23.9, 27.8)
Cambodia	2.0 (1.4, 2.6)	47.8 (43.2, 52.4)	37.5 (34.9, 40.1)	12.7 (10.1, 15.4)
Costa Rica	3.8 (3.0, 4.6)	30.0 (26.4, 33.6)	40.7 (38.1, 43.2)	25.5 (22.4, 28.7)
Dominica	5.0 (3.7, 6.2)	33.8 (31.0, 36.5)	41.6 (38.6, 44.6)	19.7 (17.2, 22.1)
Dominican Republic	2.3 (1.4, 3.3)	21.7 (18.6, 24.8)	47.6 (44.1, 51.1)	28.4 (24.4, 32.4)
Egypt	3.9 (2.1, 5.6)	32.3 (26.9, 37.7)	44.4 (39.4, 49.4)	19.4 (15.0, 23.9)
El Salvador	3.6 (2.6, 4.7)	23.1 (19.4, 26.7)	40.5 (38.4, 42.6)	32.7 (29.1, 36.3)
Eswatini	4.7 (3.1, 6.3)	40.5 (36.7, 44.3)	38.5 (35.7, 41.2)	16.3 (13.1, 19.6)
Fiji	4.7 (3.4, 5.9)	25.9 (22.5, 29.4)	44.3 (41.1, 47.4)	25.1 (22.6, 27.6)
Ghana	3.3 (1.6, 5.0)	27.5 (21.1, 33.9)	47.2 (43.5, 51.0)	22.0 (16.3, 27.7)
Guatemala	5.3 (4.0, 6.7)	25.7 (22.6, 28.9)	43.3 (40.0, 46.6)	25.6 (21.2, 30.1)
Guyana	3.8 (2.7, 4.9)	25.0 (22.3, 27.7)	43.6 (40.7, 46.5)	27.6 (24.1, 31.1)
Honduras	2.5 (1.7, 3.3)	25.8 (22.3, 29.3)	46.2 (43.7, 48.6)	25.5 (21.9, 29.1)
Indonesia	6.3 (5.4, 7.1)	40.6 (38.4, 42.7)	41.1 (39.7, 42.6)	12.0 (10.6, 13.4)
Iraq	4.1 (2.8, 5.3)	31.5 (27.7, 35.2)	42.2 (39.2, 45.1)	22.3 (19.1, 25.5)
Jamaica	1.7 (0.8, 2.6)	20.1 (16.4, 23.8)	44.5 (40.7, 48.3)	33.7 (29.9, 37.5)
Kiribati	5.0 (3.7, 6.3)	48.6 (45.6, 51.6)	36.3 (33.0, 39.6)	10.1 (7.5, 12.8)
Lao People’s Democratic Republic	3.2 (1.9, 4.4)	32.0 (26.3, 37.8)	42.0 (39.1, 45.0)	22.8 (17.7, 27.8)
Lebanon	3.2 (2.5, 3.9)	21.3 (19.3, 23.3)	46.6 (44.6, 48.6)	29.0 (26.6, 31.4)
Liberia	5.2 (3.2, 7.3)	42.1 (36.8, 47.3)	40.0 (36.1, 43.8)	12.7 (9.5, 15.9)
Malaysia	10.0 (9.2, 10.8)	42.4 (41.0, 43.8)	36.0 (34.9, 37.2)	11.6 (10.8, 12.4)
Maldives	3.4 (2.2, 4.6)	48.5 (44.7, 52.3)	35.6 (32.4, 38.8)	12.5 (10.3, 14.7)
Mauritania	3.6 (2.0, 5.2)	29.2 (24.8, 33.6)	44.6 (40.7, 48.4)	22.6 (18.0, 27.2)
Mongolia	5.4 (4.5, 6.4)	37.4 (33.5, 41.4)	42.1 (39.2, 45.1)	15.0 (12.7, 17.2)
Morocco	9.1 (8.0, 10.2)	35.0 (32.6, 37.4)	43.3 (41.3, 45.4)	12.5 (10.5, 14.5)
Mozambique	3.9 (2.4, 5.4)	24.1 (17.9, 30.3)	42.6 (34.4, 50.8)	29.4 (19.7, 39.2)
Namibia	3.9 (2.3, 5.5)	33.6 (30.1, 37.1)	44.4 (41.4, 47.5)	18.1 (15.5, 20.6)
Nepal	1.0 (0.5, 1.5)	21.9 (17.9, 25.9)	53.8 (50.0, 57.5)	23.3 (19.8, 26.8)
Pakistan	2.8 (2.1, 3.5)	56.2 (50.5, 61.9)	31.8 (27.6, 36.0)	9.2 (6.8, 11.6)
Paraguay	5.2 (4.0, 6.4)	26.4 (22.7, 30.1)	42.7 (40.2, 45.1)	25.7 (22.5, 28.9)
Peru	1.5 (0.9, 2.1)	29.9 (26.9, 32.9)	42.1 (39.9, 44.3)	26.5 (23.8, 29.1)
Philippines	7.4 (6.0, 8.7)	36.5 (32.5, 40.4)	40.9 (37.8, 44.0)	15.3 (12.9, 17.7)
Samoa	4.5 (3.3, 5.7)	28.0 (25.4, 30.5)	46.1 (41.9, 50.4)	21.4 (18.6, 24.2)
Solomon Islands	9.1 (5.5, 12.7)	32.0 (28.0, 36.1)	45.0 (39.6, 50.3)	13.9 (9.9, 17.9)
Sri Lanka	9.6 (7.1, 12.1)	46.0 (41.5, 50.5)	33.8 (29.2, 38.4)	10.5 (8.9, 12.2)
Sudan	5.7 (4.0, 7.5)	45.3 (40.9, 49.7)	35.1 (31.6, 38.6)	13.8 (10.9, 16.8)
Suriname	2.6 (1.8, 3.3)	17.1 (14.8, 19.4)	43.9 (40.8, 46.9)	36.4 (33.1, 39.8)
Syrian Arab Republic	4.5 (3.4, 5.7)	45.4 (41.6, 49.2)	37.5 (33.6, 41.3)	12.6 (10.7, 14.5)
Thailand	2.7 (1.9, 3.5)	18.4 (17.0, 19.8)	48.0 (45.5, 50.6)	30.8 (27.5, 34.2)
Timor-Leste	3.0 (1.9, 4.1)	25.7 (22.8, 28.6)	45.3 (43.6, 46.9)	26.0 (22.4, 29.7)
Tonga	6.9 (5.6, 8.2)	24.6 (22.5, 26.7)	45.1 (42.8, 47.3)	23.5 (21.6, 25.4)
Tuvalu	5.5 (4.0, 7.6)	39.7 (36.0, 43.6)	40.8 (37.0, 44.7)	14.0 (11.5, 16.9)
Tanzania	9.2 (7.3, 11.1)	44.1 (40.6, 47.6)	36.5 (33.0, 40.0)	10.2 (8.2, 12.3)
Vanuatu	12.5 (10.0, 14.9)	40.8 (37.2, 44.4)	36.6 (33.1, 40.1)	10.1 (7.6, 12.6)
Vietnam	7.6 (5.0, 10.3)	51.8 (47.8, 55.7)	32.0 (27.6, 36.4)	8.6 (6.3, 11.0)
Yemen	5.9 (3.9, 7.8)	49.9 (42.4, 57.5)	32.2 (27.3, 37.1)	12.0 (7.4, 16.6)
Pooled estimate	4.6 (4.0, 5.2)	33.3 (30.8, 35.9)	41.8 (40.7, 43.0)	20.0 (18.1, 22.0)
I^2^ (%)	94.5	97.7	90.8	97.1

The aggregated percentage within a country may not account for 100% due to the rounding error effect, which slightly affected the pooled estimate across the low-income and middle-income countries.

## Supplementary Materials

The following are available online at https://www.mdpi.com/1660-4601/17/18/6806/s1, Figure S1: Study flowchart; Table S1: Subgroup analyses for number of unhealthy eating habits.
