# Supplementary material for: Clustering of Poor Dietary Habits among Adolescents Aged 12 to 15 Years in 52 Low-Income and Middle-Income Countries
Source: Int J Environ Res Public Health. 2020 Sep 18;17(18):6806. doi: 10.3390/ijerph17186806 (PMC7558942; doi:10.3390/ijerph17186806)
Supplement: Supplementary file 1 [file ijerph-17-06806-s001.pdf]

Supplementary Material

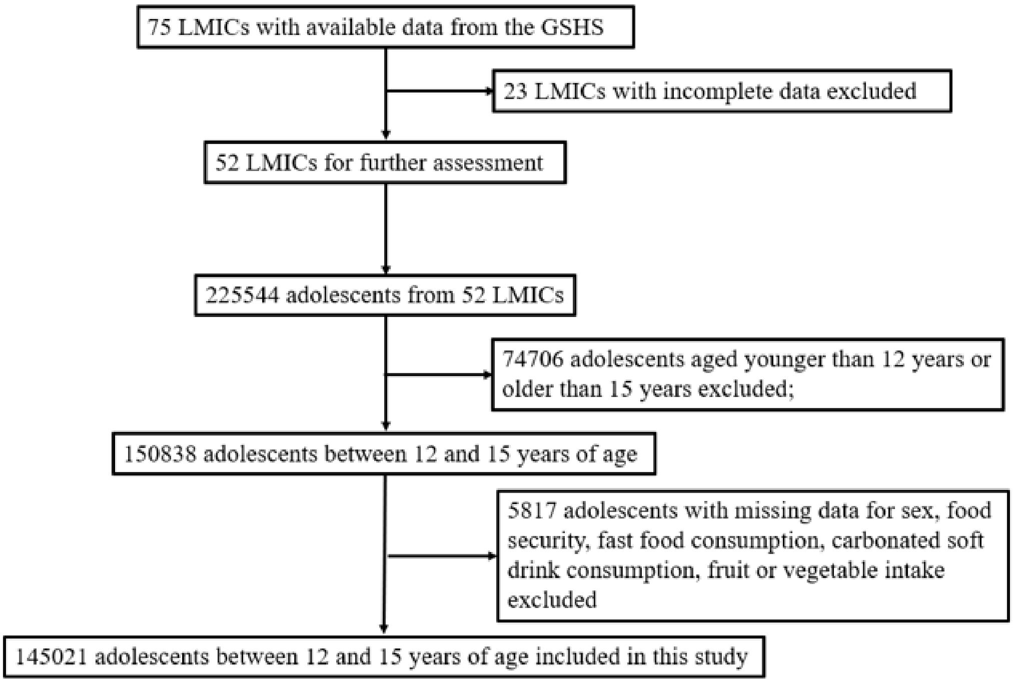

Figure S1. Study flowchart.

Table S1. Subgroup Analyses for Number of Unhealthy Eating Habits.

	No.	0		1		2		3	
		**Pooled Estimate**	**I^2**	**Pooled Estimate**	**I^2**	**Pooled Estimate**	**I^2**	**Pooled Estimate**	**I^2**
**Survey year**									
> 2013	25	4.8% (3.8%, 5.8%)	95.4%	31.7% (27.8%, 35.6%)	97.8%	42.4% (40.8%, 43.9%)	87.6%	20.9% (17.7%, 24.1%)	97.4%
⩽2013	27	4.3% (3.6%, 5.1%)	93.6%	34.8% (31.6%, 38.0%)	97.2%	41.4% (39.7%, 43.1%)	92.4%	19.2% (16.7%, 21.7%)	96.8%
**Region**									
Eastern Mediterranean	9	4.6% (3.2%, 6.0%)	93.5%	38.3% (30.9%, 45.7%)	97.6%	40.0% (36.4%, 43.5%)	91.7%	17.1% (12.4%, 21.7%)	96.5%
Africa	9	4.5% (3.4%, 5.7%)	83.1%	34.4% (28.7%, 40.2%)	95.7%	42.2% (39.2%, 45.1%)	86.9%	18.3% (14.4%, 22.1%)	93.3%
Americas	14	3.5% (2.9%, 4.2%)	88.5%	25.7% (23.0%, 28.3%)	93.7%	43.5% (42.2%, 44.8%)	78.7%	27.2% (24.2%, 30.1%)	93.9%
South-East Asia	7	3.8% (2.0%, 5.5%)	96.2%	33.6% (23.9%, 43.3%)	98.9%	42.9% (39.1%, 46.7%)	94.2%	19.5% (14.2%, 24.9%)	97.4%
Western Pacific	13	6.3% (4.7%, 8.0%)	96.3%	37.5% (32.5%, 42.5%)	97.4%	40.3% (38.0%, 42.7%)	89.6%	15.6% (12.7%, 18.6%)	96.1%
**Food security***									
Yes	31	4.1% (3.5%, 4.8%)	92.2%	32.0% (28.8%, 35.1%)	97.1%	42.1% (40.6%, 43.6%)	89.8%	21.5% (18.7%, 24.3%)	97.1%
No	21	5.2% (4.0%, 6.4%)	96.1%	35.3% (31.0%, 39.6%)	98.3%	41.4% (39.7%, 43.2%)	90.0%	17.8% (15.1%, 20.5%)	96.9%

* Country-specific food security was defined that at least 50% of participants in each country never went hungry during the past 30 days.
